# Patterns for Patient Engagement with the Hypertension Management and Effects of Electronic Health Care Provider Follow-up on These Patterns: Cluster Analysis

**DOI:** 10.2196/25630

**Published:** 2021-09-28

**Authors:** Dan Wu, Jiye An, Ping Yu, Hui Lin, Li Ma, Huilong Duan, Ning Deng

**Affiliations:** 1 College of Biomedical Engineering and Instrument Science Ministry of Education Key Laboratory of Biomedical Engineering Zhejiang University Hangzhou China; 2 School of Computing and Information Technology Faculty of Engineering and Information Sciences University of Wollongong Wollongong Australia; 3 General Hospital of Ningxia Medical University Yinchuan China

**Keywords:** hypertension, health care services, mHealth, patient engagement, electronic follow-up, cluster analysis

## Abstract

**Background:**

Hypertension is a long-term medical condition. Electronic and mobile health care services can help patients to self-manage this condition. However, not all management is effective, possibly due to different levels of patient engagement (PE) with health care services. Health care provider follow-up is an intervention to promote PE and blood pressure (BP) control.

**Objective:**

This study aimed to discover and characterize patterns of PE with a hypertension self-management app, investigate the effects of health care provider follow-up on PE, and identify the follow-up effects on BP in each PE pattern.

**Methods:**

PE was represented as the number of days that a patient recorded self-measured BP per week. The study period was the first 4 weeks for a patient to engage in the hypertension management service. K-means algorithm was used to group patients by PE. There was compliance follow-up, regular follow-up, and abnormal follow-up in management. The follow-up effect was calculated by the change in PE (CPE) and the change in systolic blood pressure (CSBP, SBP) before and after each follow-up. Chi-square tests and *z* scores were used to ascertain the distribution of gender, age, education level, SBP, and the number of follow-ups in each cluster. The follow-up effect was identified by analysis of variances. Once a significant effect was detected, Bonferroni multiple comparisons were further conducted to identify the difference between 2 clusters.

**Results:**

Patients were grouped into 4 clusters according to PE: (1) PE started low and dropped even lower (PELL), (2) PE started high and remained high (PEHH), (3) PE started high and dropped to low (PEHL), and (4) PE started low and rose to high (PELH). Significantly more patients over 60 years old were found in the PEHH cluster (*P*≤.05). Abnormal follow-up was significantly less frequent (*P≤*.05) in the PELL cluster. Compliance follow-up and regular follow-up can improve PE. In the clusters of PEHH and PELH, the improvement in PE in the first 3 weeks and the decrease in SBP in all 4 weeks were significant after follow-up. The SBP of the clusters of PELL and PELH decreased more (–6.1 mmHg and –8.4 mmHg) after follow-up in the first week.

**Conclusions:**

Four distinct PE patterns were identified for patients engaging in the hypertension self-management app. Patients aged over 60 years had higher PE in terms of recording self-measured BP using the app. Once SBP reduced, patients with low PE tended to stop using the app, and a continued decline in PE occurred simultaneously with the increase in SBP. The duration and depth of the effect of health care provider follow-up were more significant in patients with high or increased engagement after follow-up.

## Introduction

### Background

Hypertension is a lifestyle-induced chronic disease that affects health-related quality of life. Once the disease deteriorates, it may cause complications such as stroke, myocardial infarction, cardiac failure, and renal failure [1]. Controlling hypertension requires patients to follow long-term self-management plans, including measuring and recording blood pressure (BP), taking medicine, conducting physical activities, and healthy eating. These plans should preferably be established with guidance from the health care providers and be followed continuously for hypertension control [2].

The rapid pace of electronic and mobile technology development has promoted the implementation of out-of-hospital health care services [3-5]. Mobile health (mHealth) service is defined as the use of mobile phone and wireless technologies to support medical and public health care services [6]. Abundant evidence supports the introduction of mHealth services into chronic disease control and promoting positive patient behavior [4,7-10]. Although recent studies have demonstrated efficacy for some mHealth services, some have performed poorly [11-14]. Thus, there are mixed results for using mHealth services to support patient self-management of hypertension in the community [15,16].

Patient engagement (PE) is essential for bringing an improvement of health outcomes using the mHealth app [11,17,18]. PE refers to the activation of a patient to engage with the interventions that are designed to promote positive health behavior [19]. In a study of a hypertension self-management app, Toto-Ramos et al [20] found that the patients with sustained PE, as measured by the number of weeks that the patients engaged with the app, experienced significant reduction in systolic blood pressure (SBP). Goyal et al [21] evaluated the behavior of daily blood glucose reading of patients and found a significant relationship between increased number of readings and improved glycated hemoglobin. In comparison with the traditional methods, mHealth service is advantageous in improving PE for self-management of hypertension [22,23]. For example, Kaplan et al [24] designed an mHealth app to facilitate PE in managing hypertension. The app provides a mobile platform for recording and tracking self-measured BP, periodic reminders to measure BP, and wireless BP measurement devices. Their research resulted in significant improvement in BP for patients and a high level of PE.

In the mHealth field, complex PE patterns emerge when mobile apps are used for self-management. Some patients register but never use the app, some use it intermittently, and others frequently use it for a long period of time. PE can be objectively measured as amount, duration, breadth, and depth of using the mHealth app [11], and it can also be subjectively measured as interest, intrigue, focus, inattention, enjoyment, pleasure, etc [25]. In a study of pain management with a mobile app, Rahman et al [26] used a clustering technique to identify PE patterns with the app. They measured PE by 3 key usage features: duration of app use, frequency of app use, and the number of usage records. They then used a K-means clustering algorithm to find groups of patients as indicated by PE because the PE pattern reflects the behavioral characteristics of patients using the app. Sanatkar et al [27] also used a clustering technique to analyze the mHealth app usage data so as to distinguish PE with the app in an e-mental health community. They measured 5 usage features of PE: number of user logins, number of daily trackers used, number of learning activities started and completed, and number of reminders received. Distinct usage patterns were observed in the frequency of using the app.

To date, the studies on patterns of PE and the associations between these PE patterns and health outcomes have usually used various types of static count data (eg, number of logins and number of records). However, PE is a dynamic, ongoing process [28]. The change of this dynamic process cannot be captured simply by analyzing count data captured at a single date point in the cross-sectional data analysis. Longitudinal change is useful for identifying trends through analyzing the time series data. Furthermore, as hypertension management requires long-term efforts, understanding the trends of PE is important for the long-term successful management of hypertension.

Health care provider follow-up can offer continuous and personalized attention to guide patient’s self-management behavior in response to the patient’s current BP level [29]. Without health care provider support, it is difficult for patients to maintain BP control and high levels of PE with an mHealth app [30], which has often resulted in a high level of dropout [31,32]. Therefore, follow-up by health care providers is recommended by the hypertension management guideline in order to know, track, and intervene in patient’s hypertension self-management in the long-term [33-35]. In the traditional outpatient management model, follow-up often takes the form of home visits by health care providers to promote PE and then achieve BP control. Despite its proven benefits for hypertension management, follow-up is not fully implemented by health care providers due to the high human resources required for patient follow-up.

In comparison with the traditional format of follow-up, mHealth health care provider follow-up is a low-cost, convenient means of follow-up, and patient’s BP, medication, exercise, diet, etc., can be checked in the office. In a series of our previous studies into hypertension management service [36,37], after receiving the BP data that a patient entered through the mobile app, the server could automatically conduct BP data analysis, with the web-based platform reporting the patient’s BP condition to the health care provider and reminding the health care provider which patients needed to follow-up. In response to different patient health statuses, health care providers can conduct follow-up through calling and sending short SMS text messages. Our previous study designed 3 types of follow-ups: compliance follow-up, regular follow-up, and abnormal follow-up [36,37]. These were conducted to improve compliance, track BP status, redesign a new plan, and respond to the abnormal BP of patients.

To date, few studies have explored the effect of health care provider follow-up on PE and BP control in mHealth services for hypertension management [23]. It is not clear if follow-up is effective, which type of follow-up is effective, when the follow-up effect emerges, if there is a differential effect of follow-up in different patient groups, and how long the follow-up effect lasts [28,38]. Answers to these questions are essential for informing the implementation of value-based health care and providing optimal outcomes in improving the quality and reducing cost of hypertension management.

### Objective

The aim of this study was to explore the patterns of PE with a hypertension management service and the effect of health care provider follow-up on PE and BP within the first 4 weeks of mHealth app usage. This included 3 objectives: (1) to discover the patterns of PE with the mHealth app and the association between a series of related variables (age, gender, education level, the mean SBP, and the number of follow-ups) and the PE pattern, (2) to explore the health care provider follow-up effect on PE, and (3) to examine the effect of follow-up on SBP in each PE pattern.

## Methods

### Description of the mHealth App

Blood Pressure Assistant (BPA) is a mHealth hypertension self-management app available for patients in the General Hospital of Ningxia Medical University [36,37,39]. BPA was launched in 2015, and since then, 2129 patients have registered to use the app. The mHealth app was designed in accordance with a customized care pathway in compliance with the Chinese guideline for hypertension management. The care pathway defines tasks for hypertension management for patients and health care providers.

In this care pathway, each patient is required to register and provide basic demographic information (ie, name, gender, date of birth, and education level). The patient is then assigned to a health care provider, who is responsible for formulating a tailored management plan, conducting follow-up, and supervising patients’ uploaded data. The management plan includes the frequency of self-measured and recorded BP, and recommendations for medication, physical activities, and diet. The patient’s uploaded data includes self-measured BP, medications taken, physical activities, and diet records. The most important task for health care providers is to track patient’s current BP level through the web-based platform and use mobile phones for patient follow-ups to assist in BP control. There are 3 types of follow-ups. (1) Compliance follow-up is performed as a response to the identified low patient compliance with the hypertension management plan [36]. In this case, health care providers need to remind the patient to measure and record BP on a regular basis. (2) Regular follow-up is conducted on regular basis in order to check the BP level and decide whether or not to maintain or update the management plan [37]. (3) Abnormal follow-up is required when patient’s self-measured BP data are abnormal [36]. Health care providers need to check and understand the causes of abnormal conditions and intervene in a timely manner. With consent, the health care provider enrolls the patient they manage into the mHealth hypertension management program in an online community. Patients can use the app to check their self-management plans. Their main task is to measure and record BP data on a daily basis.

### Data Collection

#### Statement of Ethics

Ethical approval was granted by the Ethic Committee for the Conduct of Human Research at General Hospital of Ningxia Medical University (#NXMU-GH-2017-273). Patients in this study signed the informed consent forms.

#### Sample

All data were stored and extracted from the BPA server, which contains the demographic information, self-management plans, patient-uploaded data, and follow-up records of health care providers. The primary data set included 2129 patients. We selected the patient records based on the following criteria: patients were at least 18 years of age; patients registered to the app between March 27, 2016, and July 10, 2019 (as the main functions of the app were consistent during this period, this could ensure that the patient’s behavior was not affected by the changes in app functions); and patients continuously measured and recorded BP more than 4 weeks after registration (as the control of BP requires at least a 4-week observation period according to the relevant guidelines) [33-35].

#### Data Extraction

We extracted 3 types of data from the database: demographics, BP records, and follow-up records. The demographics information included the patient identification, data of birth, gender (male and female), and education level (primary or secondary school, high school, or university and above). The BP record data included SBP, diastolic blood pressure, and the uploaded date (containing year, month, day, minute, and second). Due to the high correlation between SBP and diastolic blood pressure, only SBP was used for analysis. The information specific to the follow-up records included patient ID, follow-up type (compliance follow-up, regular follow-up, and abnormal follow-up), and follow-up date.

### Data Analysis

#### Measuring PE

**Figure 1:**
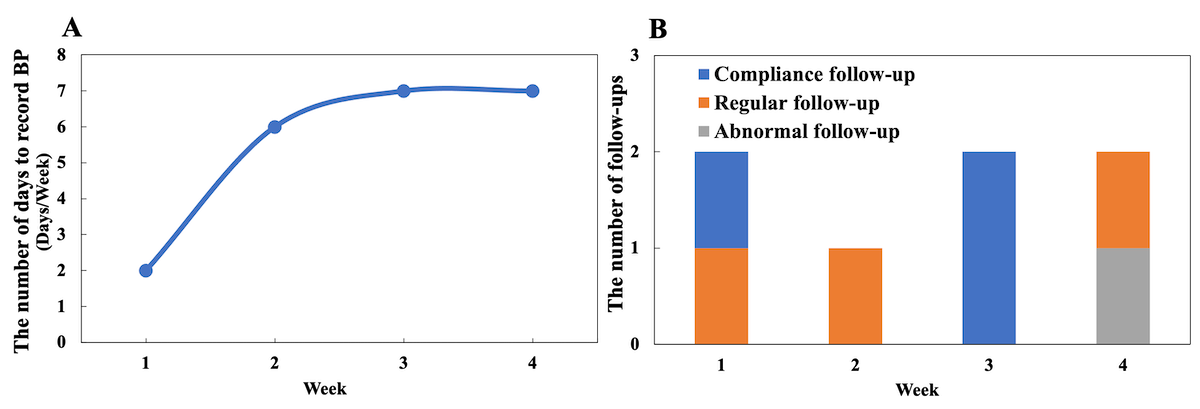
Example of patient engagement and follow-up for a patient within 4 weeks. (A) Example of patient engagement. (B) Example of the number of different types of follow-ups. BP: blood pressure.

In accordance with the hypertension management guidelines [33-35], we defined the time unit of observation as 1 week. The behavior of measuring BP is a basic behavior in hypertension management because other behaviors (eg, taking medicine, doing physical activities, and eating healthy) need to be based on patient’s current BP level. Therefore, in this study, patient engagement was indicated by the number of days that a patient recorded self-measured BP per week (see Figure 1). The analysis period was the first 4 consecutive weeks after the initial patient registration.

#### Cluster Analysis

Cluster analysis was conducted to group the patients into different PE patterns. K-means algorithm (Python 3; Python Software Foundation) was used to cluster patients by PE [40]. Dynamic time warping was used to calculate the similarity of PE between patients because this feature was represented as time series data [41]. We then used silhouette score to determine the optimal number of clusters [42]. The silhouette score measured the distance between clusters based on the distance between the PE of patients as determined by dynamic time warping. A higher silhouette score indicates tighter clusters, where each cluster is completely separate from the others.

#### Characterizing the Clusters

An optimal clustering result was reached based on the silhouette score of a different number of clusters. For each cluster, we analyzed the demographic features (the distribution of age, gender, and education level); BP features, including the distribution of mean SBP in the first week (representing the initial BP conditions in hypertension management) and the trend of weekly mean SBP; and follow-up features (the distribution of the number of the 3 types of follow-ups in each of the 4 weeks; see Figure 1).

#### The Effect of 3 Types of Follow-ups on PE and SBP

The follow-up effect was calculated by the change in PE (CPE) and the change in SBP (CSBP) before and after each follow-up, which were defined as follows:

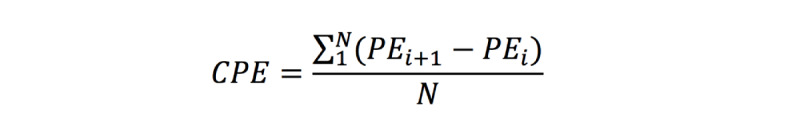

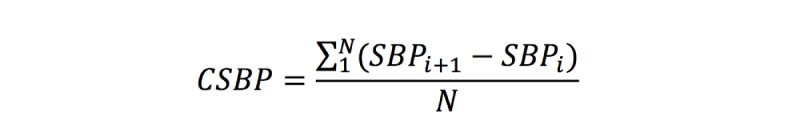





where *PE_i_*_+1_ is *PE* in the week after a follow-up event, *PE_i_* is PE in the week of the follow-up even, *SPB_i_*_+1_ is the mean SBP in the week after a follow-up event, *SPB_i_* is the mean SBP in the week of the follow-up event, and N is the number of follow-ups performed by health care providers.

Statistical analysis was conducted in SPSS version 24 (IBM Corp). Chi-square test was performed to evaluate the statistical significance of associations between the clusters and the discrete variables (demographic, BP, and the number of follow-ups). Pairwise comparisons were conducted using the *z* scores to compare difference in the proportion of discrete variables between the clusters. Analysis of variance (ANOVA) was employed to analyze the changes in outcome parameters (CPE and CSBP) after follow-up in each of the 4 weeks. Once a significant change was detected, Bonferroni multiple comparisons were further conducted to examine the differences between the clusters. A *P* value <.05 was used to determine whether the difference was statistically significant.

## Results

### PE Patterns

**Figure 2:**
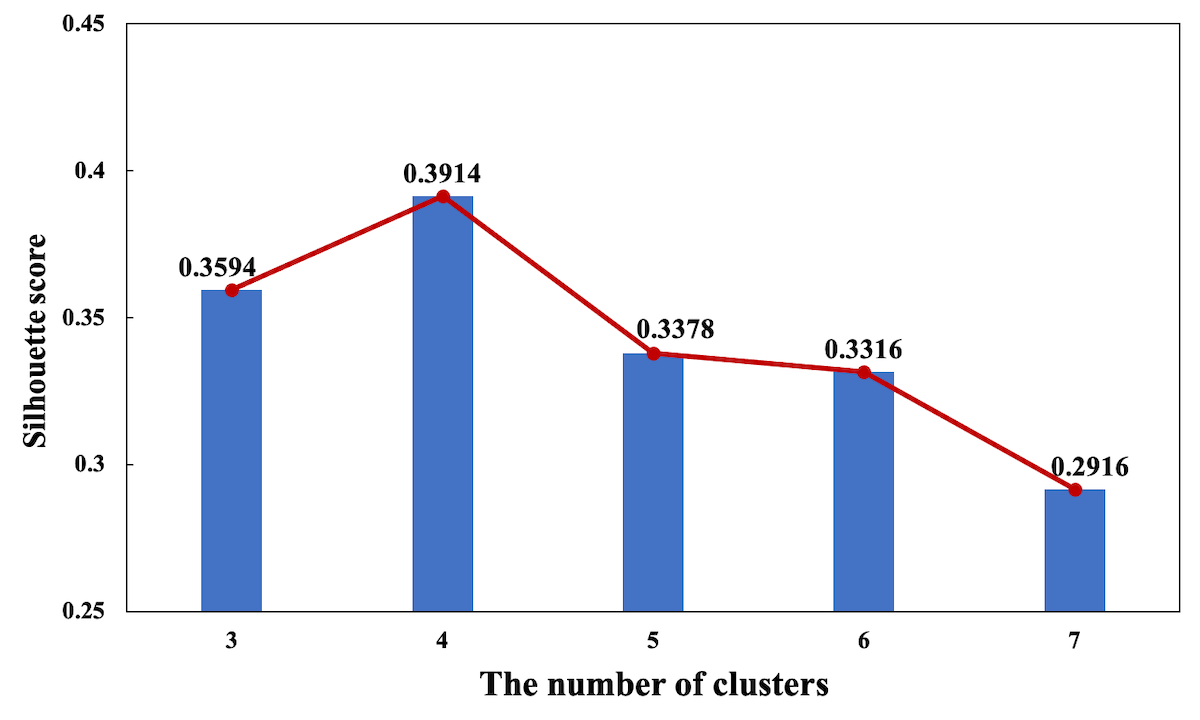
Comparison of the silhouette score for the different numberd of clusters (range from 3 to 7).

A total of 562 patients met the selection criteria and were included in the study. We found that the silhouette score was the highest with 4 clusters of patients (see Figure 2). Hence, we accepted the 4-cluster output of K-means (see Table 1) for further analysis. The PE of each cluster within 4 weeks was significantly different (*P*<.001).

**Table 1:** Comparison of PE in the 4 clusters.

PE^a^ (Days/week)	PELL^b^, mean (SD) (n=183)	PEHH^c^, mean (SD) (n=142)	PEHL^d^, mean (SD) (n=148)	PELH^e^, mean (SD) (n=89)	*P* value
Week 1	1.9 (1.1)	6.4 (1.1)	5.8 (1.2)	2.4 (1.3)	<.001
Week 2	0.8 (1.3)	6.6 (0.8)	4.8 (1.9)	3.5 (2.0)	<.001
Week 3	0.8 (1.3)	6.4 (0.9)	3.2 (2.1)	4.3 (2.0)	<.001
Week 4	0.6 (0.8)	6.3 (0.9)	1.8 (1.3)	4.6 (1.5)	<.001

^a^PE: patient engagement.

^b^PELL: patient engagement started low and dropped even lower.

^c^PEHH: patient engagement started high and remained high.

^d^PEHL: patient engagement started high and dropped to low.

^e^PELH: patient engagement started low and rose to high.

**Figure 3:**
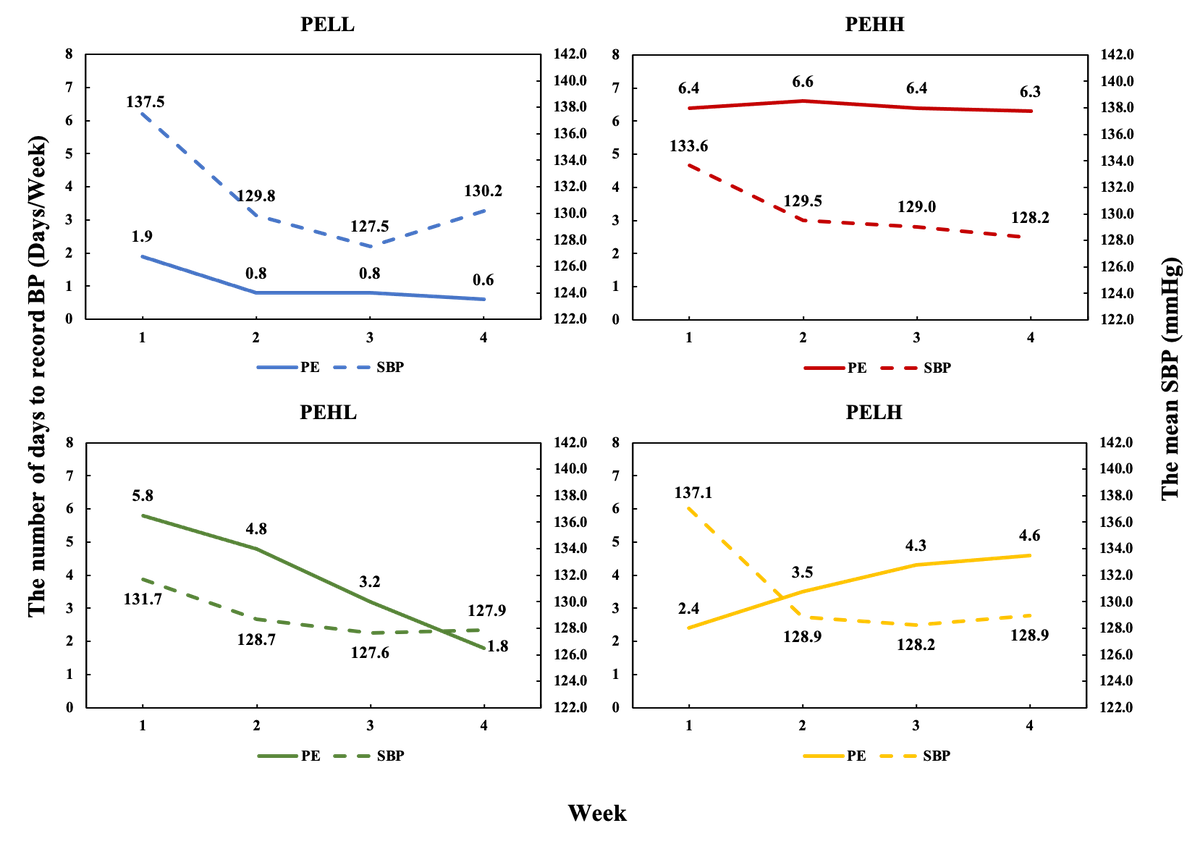
The patient engagement patterns of the 4 clusters and the 4-week mean SBP curve of the 4 clusters. BP: blood pressure; SBP: systolic blood pressure. PELL: patient engagement started low and dropped even lower; PEHH: patient engagement started high and remained high; PEHL: patient engagement started high and dropped to low; PELH: patient engagement started low and rose to high.

There were 4 distinctive change patterns of PE (see Figure 3). The first cluster contained 183 patients. They started recording their BP 1.9 days/week (SD 1.1 days/week) and then decreased every week to around 0.6 days/week (SD 0.8 d days/week) in the fourth week. We referred this cluster as “PE started low and dropped even lower” (PELL) cluster. The second cluster contained 142 patients who were consistently active engaging in BP recording throughout the whole period. On average they recorded the BP for more than 6 days per week. Therefore, we referred them as the “started high and remained high” (PEHH) cluster. The third cluster contained 148 patients who started with a high level of recording (5.8 days/week, SD 1.2 days/week) that then decreased every week to around 1.8 days/week (SD 1.3 days/week) in the fourth week. We referred them as the “PE started high and dropped to low” (PEHL) cluster. The fourth cluster contained 89 patients who began recording self-measured BP 2.4 days/week (SD 1.3 days/week) and gradually increased every week to around 4.6 days/week (SD 1.5 days/week) in the fourth week. We referred them as the “PE started low and then rose to high” (PELH) cluster.

### The Demographics, SBP, and Follow-up Characteristics of the PE Patterns

#### The Association Between Demographic Factors and the PE Pattern

The distribution of age, gender, and education level in each cluster is presented in Table 2. The majority of patients (387/562, 68.9%) were between 40 and 60 years old. Chi-square test found a statistically significant association between age and the PE pattern (*P*=.001). There was no significant difference in the distribution of patients under 60 years old in each PE pattern, but there were many more patients aged over 60 years old in the PEHH cluster than in the other clusters (*P*<.05).

Chi-square test did not find a statistically significant association between the PE pattern and gender or education level. There were many more male patients (338/562, 60.1%) using the BPA than female patients. Moreover, 50.5% (284/562) of patients had a university degree.

**Table 2:** The distribution of demographic characteristics in each cluster.

Characteristic			PELL^a^ (n=183)		PEHH^b^ (n=142)		PEHL^c^ (n=148)		PELH^d^ (n=89)	*P* value	
**Age, n (%)**											.001
	18<age≤40	24 (13.1)		14 (9.8)		27 (18.2)		11 (12.4)			
	40<age≤60	131 (71.6)		86 (60.6)		104 (70.3)		66 (74.1)			
	Age>60	28 (15.3)		42 (29.6)		17 (11.5)		12 (13.5)			
**Gender, n (%)**											.37
	Male	102 (55.7)		84 (59.2)		95 (64.2)		57 (64.0)			
	Female	81 (44.3)		58 (40.8)		53 (35.8)		32 (36.0)			
**Education, n (%)**											.45
	Primary or secondary school	14 (7.6)		6 (4.2)		5 (3.4)		2 (2.2)			
	High school	79 (43.2)		61 (43.0)		68 (45.9)		43 (48.3)			
	University and above	90 (49.2)		75 (52.8)		75 (50.7)		44 (49.4)			

^a^PELL: patient engagement started low and dropped even lower.

^b^PEHH: patient engagement started high and remained high.

^c^PEHL: patient engagement started high and dropped to low.

^d^PELH: patient engagement started low and rose to high.

#### The Association Between the Initial BP Conditions and the PE Pattern

The mean SBP in the first week represented the initial BP conditions in hypertension management. The chi-square test showed a statistically significant association between the mean SBP in the first week and the PE pattern (*P*<.001; see Table 3). *z* scores identified a significantly higher proportion of patients with high SBP (between 160 mmHg and 179 mmHg) in the PELL cluster in the first week than in the PEHL cluster (*P*<.05). The majority of patients (316/562, 56.2%) had a mean SBP between 120 mmHg and 139 mmHg in the first week.

**Table 3:** The distribution of the first week mean SBP in each cluster (*P*<.001).

SBP^a^	PELL^b^, n (%) (n=183)	PEHH^c^, n (%) (n=142)	PEHL^d^, n (%) (n=148)	PELH^e^, n (%) (n=89)
SBP<120	19 (10.4)	13 (9.2)	23 (15.5)	7 (7.9)
120≤SBP≤139	90 (49.2)	91 (64.1)	91 (61.5)	44 (49.4)
140≤SBP≤159	53 (29.0)	35 (24.6)	32 (21.6)	34 (38.2)
160≤SBP≤179	16 (8.7)	3 (2.1)	2 (1.4)	4 (4.5)
SBP≥180	5 (2.7)	0 (0)	0 (0)	0 (0)

^a^SBP: systolic blood pressure.

^b^PELL: patient engagement started low and dropped even lower.

^c^PEHH: patient engagement started high and remained high.

^d^PEHL: patient engagement started high and dropped to low.

^e^PELH: patient engagement started low and rose to high.

#### The Trend of Weekly Mean SBP in Each PE Pattern

Across 4 weeks of management, the mean SBP of all 4 clusters declined at different rates (see Figure 3). Pairwise comparisons of the mean SBP between 4 weeks in each cluster revealed that in the clusters of PELL (*P*=.002), PEHH (*P*=.006), and PELH (*P*<.001), the mean SBP had significantly reduced from the second week. In the PEHL cluster, the significant reduction of SBP appeared from the third week (*P*=.02). The PELL cluster experienced a greater reduction in the mean SBP in the third week compared to the other clusters, but the SBP of users in this cluster increased more than did the others in the fourth week.

#### The Number of Follow-ups in Each PE Pattern

The amount of the 3 types of follow-ups were significantly different (*P*=.03) in the 4 clusters (see Table 4). Abnormal follow-up was performed significantly less frequently (*P*<.05) in the PELL cluster than in the clusters of PEHH and PEHL. The distribution of follow-up in each of the 4 weeks was not significantly different in the 4 clusters. Most (338/680, 49.7%) follow-ups were performed in the first week.

**Table 4:** The distribution of the number of 3 types of follow-ups and the number of follow-ups in each of the 4 weeks in each cluster.

Value		PELL^a^ (n=183)	PEHH^b^ (n=142)	PEHL^c^ (n=148)	PELH^d^ (n=89)	*P* value	
**Type, n (%)**							.02
	Compliance	77 (46.1)	53 (31.4)	74 (39.2)	57 (41.9)		
	Regular	80 (47.9)	87 (51.5)	85 (45.0)	61 (44.9)		
	Abnormal	10 (6.0)	29 (17.2)	30 (15.9)	18 (13.2)		
**Time, n (%)**							.11
	Week 1	90 (51.7)	86 (50.3)	96 (49.2)	66 (47.1)		
	Week 2	50 (28.7)	44 (25.7)	40 (20.5)	36 (25.7)		
	Week 3	13 (7.5)	14 (8.2)	23 (11.8)	23 (16.4)		
	Week 4	21 (12.1)	27 (15.8)	36 (18.5)	15 (10.7)		

^a^PELL: patient engagement started low and dropped even lower.

^b^PEHH: patient engagement started high and remained high.

^c^PEHL: patient engagement started high and dropped to low.

^d^PELH: patient engagement started low and rose to high.

### The Effect of Follow-up on PE and SBP in Each PE Pattern

#### The Effect of Each Type of Follow-up on PE

The results of ANOVA revealed the significant main effects of cluster (*F*=15.09; *P*<.001) and type (*F*=5.442, *P*=.005), and the cluster–type interaction effect (*F*=2.60; *P*=0.02) on PE (see Table 5). Bonferroni multiple comparisons found that after the 3 types of follow-up, the CPE between the 4 clusters had the following statistically significant differences: (1) for the compliance follow-up, PE in the PELL cluster had a significantly larger drop than did the PEHH (*P*=.02) and PELH (*P*<.001) clusters, and improvement in PE in the PELH cluster was significantly higher than that of the PELL (*P*<.001) and PEHL (*P*<.001) clusters; (2) for the regular follow-up, PE in the PEHL cluster had a significantly larger drop than did the clusters of PELL (*P*=.01), PEHH (*P*=.01), and PELH (*P*<.001); and (3) for the abnormal follow-up, the decrease in PE in the PEHL cluster was significantly lower than that of the PEHH cluster (*P*=.01). Compliance follow-up improved PE in the PEHH and PELH clusters. Regular follow-up improved PE in the PELH cluster.

**Table 5:** The effect of 3 types of follow-ups on patient engagement and the effect of follow-up in each of the 4 weeks on PE. Multiple comparisons of the 4 clusters (at the .05 level).

CPE^a^ (Days/week)		PELL^b^_,_ mean (SD) (n=183)	PEHH^c^_,_ mean (SD) (n=142)	PEHL^d^_,_ mean (SD) (n=148)	PELH^e^_,_ mean (SD) (n=89)	*P* value
**Type**						
	Compliance	–0.6 (1.7)	0.5 (1.5)^f^	–0.3 (2.5)	1.31 (2.3)^f,h^	<.001
	Regular	–0.1 (1.2)	–0.2 (1.4)	–1.1 (2.3)^f,g^	0.67 (2.7)^h^	<.001
	Abnormal	–0.1 (1.0)	–0.1 (1.4)	–1.8 (2.3)^g^	–0.39 (2.3)	.01
**Time**						
	Week 1	–0.7 (1.6)	0.3 (1.7)^f^	–0.8 (2.3)^g^	1.2 (2.5)^f,g,h^	<.001
	Week 2	0.1 (1.2)	0 (0.9)	–1.5 (2.7)^f,g^	1.4 (2.1)^f,g,h^	<.001
	Week 3	–0.2 (1.3)	0.4 (1.0)	–1.6 (2.1)^g^	0.3 (2.5)^h^	.01
	Week 4	–0.2 (1.4)	–0.7 (1.7)	–0.1 (2.7)	–1.5 (2.5)	.10

^a^CPE: change in patient engagement.

^b^PELL: patient engagement started low level and dropped even lower.

^c^PEHH: patient engagement started high and remained high.

^d^PEHL: patient engagement started high and dropped to low.

^e^PELH: patient engagement started low and rose to high.

^f^The given cluster is significantly different from the PELL cluster.

^g^The given cluster is significantly different from the PEHH cluster.

^h^The given cluster is significantly different from the PEHL cluster.

#### The Effect of Follow-up on PE in Each of the 4 Weeks

The results of ANOVA revealed the significant main effects of the cluster (*F*=10.335; *P*<.001) and time (*F*=2.870; *P*=.04), and the cluster–time interaction effect (*F*=5.168; *P*<.001) on PE (see Table 5). Bonferroni multiple comparisons found that after the follow-up in each of the 4 weeks, the CPE between the 4 clusters had the following statistically significant differences: in week 1, the improvement in PE in the PELH cluster was significantly higher than that of the clusters of PELL (*P*<.001), PEHH (*P*=.03), and PEHL (*P*<.001); in week 2, the PE in the PEHL cluster had a significantly larger drop than that of the clusters of PELL (*P*=.001), PEHH (*P*=.01), and PELH (*P*<.001), and the improvement in PE in the PELH was significantly higher than that of the clusters of PELL (*P*=.01), PEHH (*P*=.01), and PEHL (*P*<.001); and in week 3, the PE in the PEHL cluster had a significantly larger drop than that of the PEHH (*P*=.02) and PELH (*P*=.01) clusters. Follow-up improved PE in the PEHH and PELH clusters in first 3 weeks and only in the second week in the PELL cluster, but had no effect on the PEHL cluster in any of the 4 weeks.

#### The Effect of Each Type of Follow-up on SBP

The results of ANOVA revealed significant main effects of cluster (*F*=2.789; *P*=.04) and type (*F*=1.137; *P*=.32), but no cluster–type interaction (*F*=0.956; *P*=.46) effect on SBP (see Table 6). The mean SBP of all 4 clusters of patients reduced after 3 types of follow-ups except for the abnormal follow-up in the PELL cluster. Bonferroni multiple comparisons showed that after the compliance follow-up, the PELH cluster had a significantly higher level of SBP decline than did the PEHL cluster (*P*=.003).

**Table 6:** The effect of 3 types of follow-ups on systolic blood pressure and the effect of follow-up in each of the 4 weeks on systolic blood pressure. Multiple comparisons of the 4 clusters (at the .05 level).

CSBP^a^ (mmHg)			PELL^b^, mean (SD) (n=183)		PEHH^c^, mean (SD) (n=142)		PEHL^d^, mean (SD) (n=148)		PELH^e^, mean (SD) (n=89)		*P* value
**Type**											
	Compliance	–3.4 (11.4)		–2.5 (5.8)		–0.9 (6.2)		–6.7 (10.5)^f^		.01	
	Regular	–3.7 (11.3)		–2.1 (6.4)		–2.4 (9.1)		–4.5 (8.7)		.33	
	Abnormal	1.8 (8.5)		–2.6 (6.3)		–1.7 (7.2)		–3.1 (11.0)		.66	
**Time**											
	Week 1	–6.1 (12.7)		–3.0 (6.5)		–2.5 (7.1)		–8.4 (9.8)^f,g^		<.001	
	Week 2	–2.1 (7.7)		–1.3 (5.2)		–0.1 (5.5)		–4.2 (9.0)		.09	
	Week 3	1.4 (13.5)		–3.2 (6.3)		2.2 (8.9)		–0.3 (10.3)		.04	
	Week 4	2.6 (6.2)		–1.7 (6.3)		–3.7 (10.7)		–3.4 (4.8)		.28	

^a^CSBP: change in systolic blood pressure

^b^PELL: patient engagement started low and dropped even lower.

^c^PEHH: patient engagement started high and remained high.

^d^PEHL: patient engagement started high and dropped to low.

^e^PELH: patient engagement started low and rose to high.

^f^The given cluster is significantly different from the PEHL cluster.

^g^The given cluster is significantly different from the PEHH cluster.

#### The Effect of Follow-up on SBP in Each of the 4 Weeks

The results of ANOVA revealed significant main effects of cluster (*F*=2.697; *P*=.045) and time (*F*=7.561; *P*<.001), but no cluster–time interaction effect (*F*=1.600; *P*=.11) on SBP (see Table 6). Bonferroni multiple comparisons showed that the PELH cluster in the first week had a significantly higher level of SBP decline than did the PEHH (*P*=.001) and PEHL (*P*<.001) clusters. The SBP decreased within the first 2 weeks in all 4 clusters and continued to fall in the PEHH and PELH clusters over 4 weeks. Overall, the SBP was more reduced in the PELL (–6.1 mmHg) and PELH (–8.4 mmHg) clusters than in the others in the first week.

## Discussion

### Principal Findings

#### PE Patterns

This study explored patterns of PE with the hypertension self-management app and identified the effect of health care provider follow-up on PE and SBP in each PE pattern in the first 4 weeks after registration. For the first time, we found 4 dynamic trends of PE in a sample of 562 patients who used the mHealth app to record self-measured BP. Two clusters started with a high level of engagement, with one remaining at a high level throughout and the other dropping. Two clusters started with low engagement, with one increasing the level of engagement and the other dropping.

The majority of patients (387/562, 68.9%) were mainly between 40 and 60 years old. There was no difference in the distribution of patients under 60 years of age in each PE cluster; however, there were more patients aged over 60 years in the continuously high engagement cluster than in the other clusters. This may suggest that the patients over 60 years of age were more likely to engage in recording self-measured BP using the mHealth app for hypertension self-management. This may be attributed to the high level of awareness of the risk of hypertension because age is an important contribution factor to the development of hypertension [16,43]. Our finding is consistent with that of Goyal et al [15] in which older participants completed more planning challenges for chronic disease management than did younger participants. However, the finding of Kruse et al [10] was not consistent with our observations. They found that patients over the age of 65 years were less likely to use the mHealth service due to problems understanding the information, difficulty using technology, and inability to access the internet [10].

In this study, there were more male patients than female patients, which may be attributed to males being more prone to using mHealth services for hypertension management [44,45]. However, we did not find a significant difference in the proportion of males and females in each PE pattern. It appears that, although more males used the mHealth services than did females [44], they had the same level of PE with the mHealth service. Abd-alrazaq et al [30] found that gender did not affect intention to use the mHealth service, which was in line with our findings. However, Chung et al [46] found that females with heart failure were more adherent to the sodium-restricted diet than males, and Goyal et al [15] found that PE levels among female users with chronic disease were slightly higher than those among male users. Therefore, the gender differences in PE with mHealth apps does not appear to be conclusive.

The relationship between PE and SBP in the PELL cluster is worth noting. Patients in the PELL cluster reported a higher SBP in the first week, and after 3 weeks of management, the SBP decreased more compared with the other clusters. Patients then began to stop using the app, and this decline in PE continued simultaneously with the increase in SBP. It may be the case that patients felt they reached the BP goal and ceased use of the app [27,47]. This may give evidence to suggest that controlling BP requires continuous engagement in hypertension self-management [35].

#### The Effect of 3 Types of Follow-ups on PE and SBP

Health care provider follow-up is essential for the prevention and treatment of hypertension. It is necessary to understand which type of follow-up is effective for which patient group [2]. Compliance follow-up, which was provided when the patients showed signs of reducing compliance in recording self-measured BP, had a positive effect on PE in the PEHH and PELH clusters. Regular follow-up, which was provided at a fixed interval, only had a positive effect on PE in the PELH cluster. These follow-ups might have been taken as the cue for engagement behavior for these patients, who may have awareness of hypertension self-management, but need cues for action. Interestingly, abnormal follow-up had no effect on any cluster of patients despite it being performed when the patient’s BP was abnormal. These findings suggest that different types of follow-ups had different effects on patients’ behavior of recording self-measured BP.

The content and method of follow-up are important to achieving a follow-up effect [48-50]. One study showed that health care providers have individual inherent preferences for the type of follow-up content to be provided to the patient [18]. Abd-alrazaq et al [30] found that the health care provider factor was related to patients’ intension to engage in mHealth services. In our study, follow-up was performed through the mobile phone after health care providers received a reminder from the mHealth app. Other ways to improve PE, such as routine reminders combined with games, have yielded higher user engagement [16], and users seem to prefer simple, short voice messages over text messages because of communication trust and increased accessibility [51]. Cechetti et al [52] developed and implemented an mHealth app with a gamification method for hypertension management, which proved to be effective in promoting PE [52].

The mean SBP of all 4 clusters of patients reduced after 3 types of follow-ups except for abnormal follow-up in the PELL cluster. We also found abnormal follow-up was performed less frequently in the PELL cluster than in the others. This may be attributed to patients in the PELL cluster having a low level of engagement in recording self-measured BP, which resulted in the mHealth app not detecting the patient’s abnormal BP condition and reminding the health care provider to follow-up. The PELH cluster developed positive PE and achieved SBP control after 3 types of follow-ups. This is in agreement with the observation that follow-up can motivate patients to engage in self-management. Positive feedback from PE after follow-up has been shown to be beneficial for BP control [53-55].

#### The Effect of Follow-up in Each of the 4 Weeks on PE and SBP

One study reported that 74.84% of app-only users stopped using an mHealth physical activity management app by day 43 [56]. This suggests that the duration of patient “stickiness” with the fully automatic mHealth services is limited and needs to be complemented by human support to keep momentum. Little is still known concerning the extent and duration of the effect of health care provider follow-up on self-management of hypertensive patients. This study found that the effect of follow-up on the PEHH and PELH clusters lasted until the third week, only had a small impact on the PELL cluster in the second week, and had no effect on the PEHL cluster at any time point. The effect duration of different types of follow-ups varied across each PE pattern. This may suggest that a fixed care pathway based only on patient’s BP level would not work for all types of patients. Chronic disease management also needs to consider the patient’s behavior and personal preferences. Our results support the design of patient-centered follow-up plans that incorporate social behavioral characteristics and preferences of patients into chronic disease management [14].

After 4 weeks of mHealth services being used, the mean SBP of all 4 clusters decreased. Interestingly, in the first week, the PELL and PELH clusters had a higher level of decline in SBP than did the other clusters. This may be the reason why the PELL cluster was complacent and did not actively engage in recording self-measured BP. Taking the advice of the health care providers may motivate those in the PELH cluster to increase their level of engagement. In the first 2 weeks, all 4 clusters of patients experienced a decline in SBP. Only the PEHH and PELH clusters maintained the SBP reduction through the fourth week, supporting the notion that hypertension management requires ongoing effort in monitoring BP to help patients improve their awareness of their own condition.

### Strengths and Limitations

The strength of this study was that, first, the study used the longitudinal data collected in the first 4 weeks of patient registration with the mHealth service, which is informative for characterizing the changing trend of PE with hypertension management under an mHealth service. The findings are useful for continuous improvement of mHealth services for hypertension management. To the best of our knowledge, no other study has used longitudinal data to describe patterns of PE with a hypertension self-management app. Second, we analyzed the effect of health care provider follow-up on PE and SBP in each PE pattern. Our findings revealed the relationship between PE, BP, and health care provider follow-up. This provides evidence to support the further design of appropriate types of follow-ups for patients. Finally, we observed PE from real-world patients, which can reveal patient behavior in a natural setting, rather than recruited patients who would be more likely to overcome the burden associated with research work [57]. This helped to generate implementable, practical insights for the engagement of actual patients in daily hypertension management.

The study had 4 limitations. First, we excluded patients who used the app for fewer than 4 weeks after registration. This might have produced a bias toward more positive findings of PE with the mHealth app and thus may limit the generalizability of our findings to those patients who drop out of the mHealth service early. Second, we only investigated the PE trend with the mHealth service in the first 4 weeks of hypertension management. Future study can further investigate the PE pattern after this period of time. Third, we defined PE as the behavior of recording self-measured BP. There are many other usage behaviors, such as taking medicine, engaging in physical activities, and keeping a healthy diet, which should be analyzed in future studies. Fourth, the different engagement behaviors could have arisen from various demographic and social psychological characteristics of patients (such as marital status, profession, anxiety, depression), so these factors need to be considered in future studies.

### Conclusions

By analyzing the 4-week log data from a hypertension self-management app, BPA, this study identified the 4 distinct PE patterns in using an mHealth app for hypertension self-management. We also characterized the different effects of 3 types of health care provider follow-up on PE and SBP. Results showed how patients engaged with the mobile app and how health care provider follow-up affects or does not affect their engagement and BP. Our findings may inform the design and help strengthen health care provider follow-up strategies to improve outpatient engagement with mHealth apps for hypertension management. Future work needs to clarify the long-term engagement of patients with hypertension health care services. The indicators of PE should be broadened to include multiple types of usage behavior, and the effect of patient provider follow-up needs to be associated with patients’ various demographic and socio-psychological characteristics.

## Abbreviations

ANOVA: analysis of variance
BP: blood pressure
BPA: Blood Pressure Assistant
CPE: change in patient engagement
CSBP: the change in systolic blood pressure
mHealth: mobile health
PE: patient engagement
PELL: patient engagement started low and dropped even lower
PEHH: patient engagement started high and remained high
PEHL: patient engagement started high and dropped to low
PELH: patient engagement started low and rose to high
SBP: systolic blood pressure
